# Investigation of Toll-like receptors in the pathogenesis of fibrotic and granulomatous disorders: a bronchoalveolar lavage study

**DOI:** 10.1186/1755-1536-3-20

**Published:** 2010-10-11

**Authors:** Giorgos A Margaritopoulos, Katerina M Antoniou, Kostas Karagiannis, Katerina D Samara, Ismini Lasithiotaki, Evi Vassalou, Rena Lymbouridou, Helen Koutala, Nikos M Siafakas

**Affiliations:** 1 Interstitial Lung Disease Unit, Department of Thoracic Medicine, University Hospital of Heraklion, Crete, Greece; 2 Laboratory of Molecular and Cellular Pulmonary Medicine, Medical School, University of Crete, Greece; 3 Laboratory of Hematology, Medical School, University of Crete, Greece

## Abstract

**Background and aim:**

Toll-like receptors (TLRs), a key component of innate immunity, have recently been implicated in the pathogenesis of interstitial lung diseases (ILDs). As the involvement of TLRs has not yet been fully elucidated, the aim of the current study was to examine the expression of various TLRs in the bronchoalveolar lavage fluid (BALF) of patients with ILDs.

**Patients and Methods:**

We studied prospectively three groups of patients: (1) one group of 35 patients with fibrotic disorders, 16 with idiopathic pulmonary fibrosis (IPF) and 19 with fibrotic interstitial pneumonias associated with collagen tissue disorders (CTD-IPs); (2) one group of 14 patients with pulmonary sarcoidosis; and (3) 11 normal subjects. We evaluated TLR expression with flow cytometry and mRNA expression with real-time PCR.

**Results:**

An overexpression of TLR-3 mRNA was found in fibrotic disorders (CTD-IPs/IPF) in comparison with sarcoidosis (mean ± SD, 1.104 ± 1.087 versus 0.038 ± 0.03; *P *= 0.04). Additionally, TLR-3 mRNA was increased in CTD-IPs in comparison with IPF (*P *= 0.001), sarcoidosis (*P *= 0.002) and controls (*P *= 0.05). An upregulation in TLR-7 and -9 mRNA expression was detected in IPF (*P *= 0.05) and sarcoidosis (*P *= 0.05), respectively, when compared to controls. A higher percentage of TLR-9-expressing cells was found in BALF of CTD-IPs when compared to IPF (mean ± SD, 36.7 ± 7.06 versus 14.85 ± 3.82; *P *= 0.025).

**Conclusion:**

We observed distinct profiles of TLR expression in fibrotic and granulomatous disorders. It is likely that they could play a key role in the pathogenesis of these diseases and represent future therapeutic targets.

## Background

Interstitial lung diseases (ILDs) include a wide spectrum of disorders, many of which are uncommon and many of unknown etiology. Pathogenesis of idiopathic and autoimmune fibrotic lung diseases as well as granulomatous lung disorders still remains an enigma. Repetitive cycles of epithelial injury, fibroblast activation and abnormal wound repair are considered main events [1]. Several factors such as genetic and epigenetic abnormalities, infections, cytokines and growth factors, increased oxidative stress, autoantibodies, environmental exposures and gastroesophageal reflux have been suggested as possible contributors for the initiation and progression of ILDs [2,3].

The lungs are constantly exposed to pathogens and their byproducts and represent a frequent site of infections. Toll-like receptors (TLRs) are pattern recognition receptors that play a key role in the innate immunity, representing the first line of host defense against pathogens. TLRs localize to various cellular compartments, depending on the nature of the ligands they recognize. Thus, TLRs involved in recognition of lipid and protein ligands are expressed on the plasma membrane (TLR-1, TLR-2, TLR-4, TLR-5 and TLR-6), whereas TLRs that detect viral nucleic acids are localized in endolysosomal cellular compartments (TLR-3, TLR-7, TLR-8 and TLR-9). Endosomal TLRs recognize various conserved pathogen-associated molecular patterns (PAMPs) such as viral derived RNA (TLR-3, -7 and -8) and DNA (TLR-9), as well as endogenous ligands released following tissue damage, cell death, oxidative stress and decomposition of extracellular matrix (ECM) [4-6]. TLR expression can also be detected in type II alveolar epithelial cells, airway epithelial cells, smooth muscle cells and fibroblasts [7-10]. TLR activation induces signaling pathways leading to the expression of inflammatory mediators and induction of an immune response able to eliminate the pathogen successfully. However, should this process be ineffective, the infectious stimuli persist and provoke a sustained injury, leading to chronic inflammation and skewing of the immune response from a Th1 toward a Th2 cytokine pattern, thus facilitating the development of fibrosis [11]. This has led to the hypothesis that infectious diseases could be a cofactor in the pathogenesis of ILDs.

Novel data in this field have underlined the role of TLR-9 in pulmonary fibrosis as it was shown to be overexpressed in pulmonary fibroblasts of patients with IPF [12]. Moreover, bleomycin (BLM)-induced fibrosis may be mediated by activation of TLR-2 and TLR-2 deficiency, or treatment with a TLR-2 antagonist not only protects but also reverses BLM-induced fibrosis [13]. Infections have been implicated in the pathogenesis of sarcoidosis, since DNA from mycobacteria and propionibacteria have been found in sarcoid tissue [14,15]. A higher expression of TLR-2 and -4 has been demonstrated in peripheral blood monocytes [16], and linkage analysis has indicated that an unidentified polymorphism of TLR-4 is associated with sarcoidosis [17]. TLRs are also implicated in the pathogenesis of autoimmune disorders such as systemic lupus erythematosus [18], rheumatoid arthritis [19-21], systemic sclerosis [22], dermatomyositis and Sjögren syndrome [23].

Our aim was to investigate whether dysfunctions of the immune system at the TLR level could elucidate these pathogenetic pathways and explain differences in prognosis between fibrotic and granulomatous disorders. Toward this purpose, we assessed the percentage of TLR-expressing cells by flow cytometry (TLR-2, -4 and -9) and the mRNA expression of various TLRs (-2, -3, -4, -7, -8 and -9) in the bronchoalveolar lavage fluid (BALF) of the patient group and healthy controls.

## Patients and Methods

### Patients

Sixty (60) consecutive patients from the Interstitial Lung Disease Unit of the Department of Thoracic Medicine, University Hospital of Heraklion, were enrolled in the study: 35 patients with fibrotic disorders, 16 with idiopathic pulmonary fibrosis (IPF) and 19 with fibrotic interstitial pneumonias associated with collagen tissue disorders (CTD-IPs), 14 patients with sarcoidosis and 11 control subjects.

The ethics committee of our hospital approved the protocol, and all patients and controls gave their consent.

### Group A: Fibrotic Lung Disorders

1. *IPF: *The diagnosis was based on internationally accepted clinical and imaging criteria [24]. In six cases, diagnosis was made by video-assisted thoracoscopic surgery (VATS), where the histologic diagnosis of usual interstitial pneumonia (UIP) was obtained. In the remaining 10 cases, the diagnosis was made on the basis of clinical and high-resolution computed tomography (HRCT) criteria: (1) bilateral basal or widespread crackles; (2) restrictive ventilatory defect or isolated reduction of DL_CO_; (3) computed tomography (CT) findings indicative of IPF, i.e., predominantly basal and subpleural microcystic or macrocystic honeycombing, with variably extensive ground-glass and reticular abnormalities but no consolidation, nodular abnormalities, or other parenchymal abnormalities (apart from centrilobular emphysema); and (4) no history of environmental exposure to a fibrogenic agent or connective tissue disease [24]. According to the aforementioned criteria, any known cause of pulmonary fibrosis, such as a connective tissue disorder, was excluded by both immunologic screening and rheumatological clinical evaluation.

2. *CTD-IPs: *The diagnosis was based on clinical and HRCT criteria in accordance with the international societies' guidelines [25-27]. In detail, the following patients were studied (1) eight patients with Rheumatoid Arthritis (RA) and HRCT characteristics of UIP; (2) seven patients with systemic sclerosis (SSc) and HRCT appearance of NSIP; (3) two patients with systemic lupus erythematous (SLE), one with HRCT features of NSIP and one with HRCT features of UIP; (4) one patient with Sjögren syndrome and histologically proven fibrotic NSIP; and (5) one patient with dermatomyositis-polymyositis and HRCT features of NSIP.

### Group B: Sarcoidosis

Fourteen sarcoidosis patients were enrolled in the study. Diagnosis was made according to the ATS/ERS/World Association of Sarcoidosis and Other Granulomatous Disorders joint statement [28]. All patients had transbronchial or surgical lung biopsy with histopathological evidence of noncaseating epithelioid cell granulomas without evidence of infection or inorganic material to account for the pulmonary granulomatous reaction. According to the chest radiographic classification of sarcoidosis, three patients had stage I disease (lymphadenopathy alone), five had stage II disease (lymphadenopathy and parenchymal opacities) and six had stage III disease (only parenchymal opacities).

## Methods

### Pulmonary function tests

All patients were evaluated with complete pulmonary function tests (PFTs), including spirometry, measurement of lung volumes and diffusion capacity. Spirometry, lung volumes using the helium-dilution technique and *T*_L, CO _(corrected for hemoglobin) using the single-breath technique were performed using a computerized system (Jaeger 2.12; MasterLab, Würzburg, Germany). Predicted values were obtained from the standardized lung function testing of the European Coal and Steel Community, Luxembourg (1993).

### BALF processing

BALF was obtained from all patients as previously described [29]. Briefly, a flexible bronchoscope was wedged into a subsegmental bronchus of a predetermined region of interest based on radiographic findings. A BALF technique was performed by instilling a total of 240 mL of normal saline in 60-mL aliquots, each retrieved by low suction. The BALF fractions were pooled and split equally into two samples. One sample was sent to the clinical microbiology and cytology laboratory, and the other sample was placed on ice and transported to the research laboratory. The research sample was filtered through sterile gauze (Thompson, Ontario, Canada) and centrifuged at 400 *g *for 15 min at 4°C.

Total cell counts were determined using an improved Neubauer counting chamber and expressed as the total number of cells per millilitre of aspirated fluid. The pellet was washed three times with cold phosphate-buffered saline/Dulbecco's modified Eagle's medium, and the cells were adjusted to a final concentration of 10^6 ^cells/mL with RPMI 1640 solution plus 2% fetal calf serum. The slide preparation was performed as previously reported [30].

### Flow cytometric analysis

The samples were analyzed on an Epics Elite (Coultronics, Luton, UK) fluorescence-activated flow cytometer. The white cells were tightly gated by volume and complexity on a forward (0{FC33}°) and side light-scattering (90{FC33}°) mode and by CD45+ expression (pan leukocyte marker). A minimum of 10^5 ^cells were analyzed in each case. The appropriate control was used for subtraction of the background. The percentage of one- and two- color positive cells was measured. The following mouse antihuman monoclonal antibodies were used for labeling BALF cells: phycocyanate (Pcy-5)-conjugated anti-CD45+, phycoerythrin (PE)-conjugated anti-CD3+, fluorescein isothiocyanate (FITC)-conjugated anti-CD4+, and FITC-conjugated anti-CD8+ (Immunotech, Marseille, France). Mouse anti-mouse isotype-matched FITC-, PE- or PCy-5-conjugated immunoglobulin were used as control antibodies.

### RNA Isolation and Reverse Transcription-Polymerase Chain Reaction: RNA extraction and reverse transcription

Total RNA was extracted from each specimen using a power homogenizer and TRIzol reagent (Invitrogen, Carlsbad, CA, USA) according to the manufacturer's instructions. cDNA was synthesized using the Strascript reverse transcriptase kit (Stratagene, La Jolla, CA, USA) as previously described [31].

### Real-time RT-PCR

TLR mRNA expression was measured using a real-time RT-PCR assay with SYBR-Green I. Primers were designed to span introns. β-actin was used as the internal control to normalize TLR-2, TLR-3, TLR-4, TLR-7, TLR-8 and TLR-9 (Table 1). Specifically, 1 μl cDNA from all patient and control samples was amplified in a PCR reaction containing 2 × Brilliant SYBR-Green I QPCR Master Mix, 300 nM of each primer and 30 μM ROX passive reference dye in a final volume of 20 μl. After an initial denaturation at 95°C for 10 min, the samples were subjected to 40 cycles of amplification comprising denaturation at 95°C for 30 sec, annealing at appropriate temperature for each primer pair for 30 sec and elongation at 72°C for 30 sec, followed by a melt curve analysis, in which the temperature was increased from 55°C to 95°C at a linear rate of 0.2°C/sec. Data collection was performed both during annealing and extension, with two measurements at each step, and at all times during melt curve analysis. In each PCR reaction two nontemplate controls were included. All PCR experiments were conducted on the Mx3000P real-time PCR thermal cycler using software version 2.00 Build 215, Schema 60 (Stratagene). To verify the results of the melt curve analysis, PCR products were analyzed by electrophoresis in 2% agarose gels stained with ethidium bromide and photographed on a UV light transilluminator. Primer sequences, annealing temperatures and PCR products length for all the TLRs analyzed, as well as for β-actin are shown in Table 1. All reactions were run in triplicate, and peptide TLR transcript levels were calculated and normalized to each specimen's housekeeping gene mRNA (β-actin) as well as the appropriate calibrators using the ΔΔCt method for relative quantification. Specifically, after amplification, standard curves were constructed from samples used in a series of consecutive dilutions for both the gene of interest and the internal control (β-actin). TLRs and β-actin amplification efficiencies were the same, reaching 100%. IPF and control data were first normalized against variation in sample quality and quantity. Normalized values to β-actin, ΔCts, were initially calculated using the following equation: ΔCt_sample _= Ct_TLRs _- Ct_β_-actin.

**Table 1:** Primer sequences used for quantitative real-time RT-PCR.

Gene	Primer pair Sequence (5'-3')	Annealing temperature
*TLR-2*	For: GGGTTGAAGCACTGGACAAT	55°C
	Rev: TTCTTCCTTGGAGAGGCTGA	
*TLR-3*	For: ACACCATCTATTAAAAGACCCATTAT	62°C
	Rev: TCCAGATTTTGTTCAATAGCTTGTT	
*TLR-4*	For: GGTCACCTTTTCTTGATTCCA	55°C
	Rev: TCAGAGGTCCATCAAACATCAC	
*TLR-7*	For: GCTATCAGATTCAAAAACAACAGAA	55°C
	Rev: CACAAACACCTTTGTAGATCACTTCT	
*TLR-8*	For: GGCTTTCTTTCTGAAGTCAGTAGTCT	58°C
	Rev: TTTCCGTGTAGTTCCAACATAGATAA	
*TLR-9*	For: CTGAGTGAGAACTTCCTCTACAAATG	58°C
	Rev: TCTTTTGGTAATTGAAGGACAGGTTA	
*β-Actin*	For: CGGCATCGTCACCAACTG	60°C
	Rev: GGCACACGCAGCTCATTG	

## Statistical analysis

TLR mRNA levels and flow cytometry results were first evaluated using the one-sample Kolmogorov-Smirnov goodness of fit test to determine whether they followed a normal distribution. On the basis of the results, the nonparametric Mann-Whitney *U *test or the parametric *t*-test was used to examine correlations. A χ^2 ^test was used as indicated to examine TLR-2-3-4-7-8-9 expression status among IPF, sarcoidosis, CTD-IPs and control groups. Statistical analysis was carried out using SPSS 17.0 software (SPSS, Chicago, IL, USA). Statistical significance was set at the 95% level (*P *< 0.05).

## Results

### Demographics

Patients with fibrotic disorders were older than sarcoidosis patients. Female gender was more prevalent in the CTD-IPs and sarcoidosis group than in the IPF group. Most of the patients with CTD-IP were nonsmokers, whereas most of the patients of all other groups, including controls, had a current or past smoking history. A trend of significant lower forced vital capacity (FVC) in IPF in comparison with sarcoidosis patients has been observed. DLco levels indicated more severe disease in IPF patients compared to sarcoidosis and healthy controls, while CTD-IP patients had a significantly lower DLco than sarcoidosis (Table 2).

**Table 2:** Demographic and lung function characteristics of patient groups.

Characteristics	Controls	IPF	CTD-IP	SARC	*P *value
Number	11	16	16	14	-
Age*	58.44 ± 3.49	67.27 ± 1.62	64.23 ± 4.29	47.42 ± 4.65	p2:0.001 p3:0.04 p4:0.01
Gender: (male/female)**	8/3	12/4	3/13	5/9	p1: < 10^-4 ^p2:0.02 p5:0.002 p6:0.04
Nonsmokers**	3	6	14	5	p1: 0.00 p4:0.004 p5:0.001
Smokers**	7	1	1	4	p3:0.001 p5:0.001
Ex-smokers**	1	9	1	5	p1:0.003 p3:0.02
FEV1*	85.11 ± 7.89	82.44 ± 6.15	86.93 ± 6.37	89.83 ± 6.96	NS
FVC*	91.56 ± 7.46	76.06 ± 5.23	81.07 ± 5.65	91.75 ± 5.97	p2:0.06
FEV1/FVC*	74.56 ± 4.48	85.14 ± 2.76	85.36 ± 2.57	81.36 ± 2.63	p5:0.057
DLCO*	76.22 ± 7.72	54.19 ± 7.18	60.15 ± 5.12	91.3 ± 8.06	p2:0.002 p3:0.05 p4:0.005
KCO*	86.22 ± 8.14	87.63 ± 6.44	79 ± 6.92	108.9 ± 5.91	p2:0.02 p4:0.003 p6:0.04

Values are expressed as means ± SEM (standard error of the mean).

*t-test; *P *< 0.05 is considered statistically significant.

**χ^2 ^test; *P *< 0.05 is considered statistically significant.

NS, not significant.

Abbreviations: FEV1: forced expiratory volume in one second, FVC: forced vital capacity, TLC: total lung capacity, DL_CO_, diffusing capacity for carbon monoxide, KCO: DLCO per unit lung volume. p1: *P *value between IPF and CTD-IPs, p2: *P *value between IPF and sarcoidosis, p3: *P *value between IPF and control, p4: *P *value between CTD-IP and sarcoidosis, p5: *P *value between CTD-IP and control, p6: *P *value between sarcoidosis and control.

### Flow cytometry results

**Figure 1:**
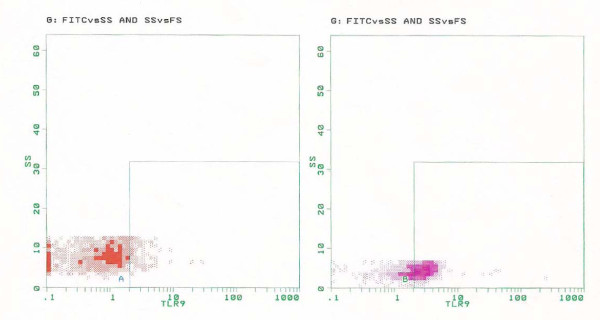
**Expression of Toll-like receptor (TLR)-9 in bronchoalveolar lavage fluid (BALF) cells in one representative patient with idiopathic pulmonary fibrosis (IPF) (right) and one with fibrotic interstitial pneumonias associated with collagen tissue disorders (CTD-IP) (left)**.

**Figure 2:**
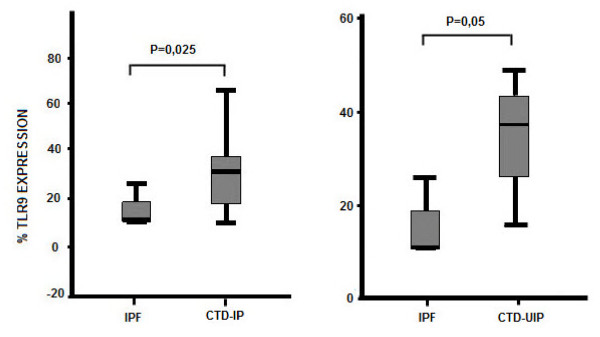
**Expression of TLR-9 in BALF cells of patients with IPF, CTD-IP and CTD-UIP**.

A statistically significant difference has been found for TLR-9 expression in BALF cells of patients with CTD-IPs when compared to IPF patients (mean ± SD, 36.7 ± 7.06 versus 14.85 ± 3.82; *P *= 0.025) (Figures 1, 2 and Table 3). Given the heterogeneity of CTD-IP group, when we compared patients with CTD-IP and HRCT features of UIP to IPF patients we found that TLR-9 expression was still significantly higher in CTD-UIP than in IPF (mean ± SD, 34.85 ± 6.92 versus 14.85 ± 3.82; *P *= 0.05) (Figure 2). No significant difference has been detected in the flow cytometry results for TLR-2 or TLR-4 among the study groups.

**Table 3:** Flow cytometry results.

	IPF	CTD-IP	Sarcoidosis	Controls	*P *value
TLR-2	18.4 ± 13.27	3.12 ± 0.96	3.23 ± 1.31	4.22 ± 1	NS
TLR-4	32.45 ± 5.41	38.52 ± 4.94	28.06 ± 6.34	29.3 ± 8.15	NS
TLR-9	14.85 ± 3.82	36.7 ± 7.06	27 ± 6.72	36.8 ± 10.26	**p1**:0.025

Values are expressed as the mean ± SEM (standard error of the mean). Mann-Whitney *U *test; *P *< 0.05 is considered statistically significant. NS, not significant. p1: *P *value between IPF and CTD-IPs, p2: *P *value between IPF and sarcoidosis, p3: *P *value between IPF and control, p4: *P *value between CTD-IP and sarcoidosis, p5: *P *value between CTD-IP and control, p6: *P *value between sarcoidosis and control.

### TLR mRNA expression

**Figure 3:**
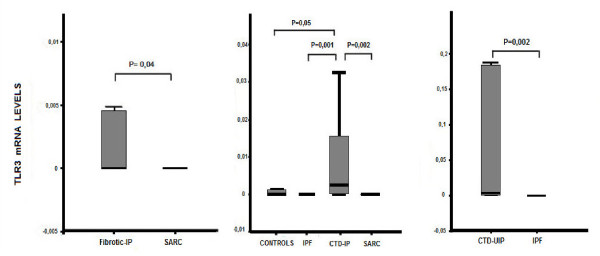
**mRNA expression of TLR-3 in patient group and healthy subjects**.

TLRs mRNA expression was detectable in patients and control groups as shown in Table 4. TLR-3 mRNA expression was significantly higher in patients with fibrotic disorders (CTD-IPs and IPF) when compared to those with granulomatous disorders (sarcoidosis) (mean ± SD, 1.104 ± 1,087 versus 0.038 ± 0.037; *P *= 0.04). In addition, TLR-3 mRNA expression was significantly higher when we compared patients with CTD-IP to IPF (mean ± SD, 2.03 ± 2 versus 0.007 ± 0.005; *P *= 0.001). When we compared patients with CTD-IP and HRCT features of UIP to IPF patients, we found that TLR-3 mRNA expression was still significantly higher in CTD-UIP than in IPF (mean ± SD, 3.847 ± 3.804 versus 0.007 ± 0.005; *P *= 0.002). Moreover, patients with CTD-IP had an increased TLR-3 mRNA expression in comparison with sarcoidosis (mean ± SD, 2.03 ± 2 versus 0.038 ± 0.037; *P *= 0.002) and control subjects (mean ± SD, 2.03 ± 2 versus 0.0014 ± 0.0008; *P *= 0.05) (Table 5 and Figure 3). Moreover, TLR-7 mRNA expression was increased in patients with IPF when compared to control group (mean ± SD, 5.06 ± 4.12 versus 1.5 ± 1.35; *P *= 0.05) (Table 5). TLR-9 mRNA expression was marginally increased in patients with sarcoidosis when compared to healthy subjects (mean ± SD, 213.52 ± 211.95 versus 0.58 ± 0.53; *P *= 0.05) (Table 5). No statistically significant difference has been detected at the mRNA expression of TLR-2-4-8 among patient groups and control subjects (Table 5).

**Table 4:** Expression profile of TLR mRNA in patient group and control subjects.

	IPF	CTD-IP	SARCOIDOSIS	CONTROLS	*P *value
TLR-2	100 (16/16)	94.7 (18/19)	100 (14/14)	88.9 (8/9)	NS
TLR-3	12.5 (2/16)	78.94 (15/19)	14.3 (2/14)	33.3 (3/9)	p1:< 10^-4 ^p4: < 10^-4^ p5:0.01
TLR-4	93.75 (15/16)	89.4 (17/19)	85.7 (12/14)	77.7 (7/9)	NS
TLR-7	93.75 (15/16)	94.7 (18/19)	100 (14/14)	77.7 (7/9)	NS
TLR-8	100 (16/16)	100 (19/19)	92.9 (13/14)	100 (9/9)	NS
TLR-9	100 (16/16)	100 (19/19)	92.9 (13/14)	77.7 (7/9)	p3:0.04 p5:0.03

χ^2 ^test; *P *< 0.05 is considered statistically significant. NS, not significant. p1: *P *value between IPF and CTD-IPs, p2: *P *value between IPF and sarcoidosis, p3: *P *value between IPF and control, p4: *P *value between CTD-IP and sarcoidosis, p5: *P *value between CTD-IP and control, p6: *P *value between sarcoidosis and control.

**Table 5:** mRNA expression of TLRs in patient group and control subjects.

	IPF	CTD-IP	Sarcoidosis	Controls	*P *value
TLR-2	9.75 ± 7.54	4.04 ± 3.53	599.9 ± 597.7	1.72 ± 1.63	NS
TLR-3	0.007 ± 0.005	2.03 ± 2	0.038 ± 0.037	0.0014 ± 0.0008	p1:0.001 p4:0.002 p5:0.05
TLR-4	2.34 ± 1.61	1.35 ± 1.12	20.98 ± 19.3	12.37 ± 12.24	NS
TLR-7	5.06 ± 4.12	12.92 ± 10.25	29 ± 26.81	1.5 ± 1.35	p3:0.05
TLR-8	11.14 ± 7.89	14.5 ± 11.65	679.33 ± 672.34	8.67 ± 8.51	NS
TLR-9	3.1 ± 2.51	1.54 ± 0.94	213.52 ± 211.95	0.58 ± 0.53	p6:0.05

Values are expressed as means ± SEM (standard error of the mean). Mann-Whitney *U *test; *P *< 0.05 is considered statistically significant. NS, not significant.

p1: *P *value between IPF and CTD-IPs, p2: *P *value between IPF and sarcoidosis, p3: *P *value between IPF and control, p4: *P *value between CTD-IP and sarcoidosis, p5: *P *value between CTD-IP and control, p6: *P *value between sarcoidosis and control.

Furthermore, we explored the impact of smoking in TLRs mRNA expression. For this reason, we divided the patients of each group (IPF, CTD-IP, sarcoidosis) into two subgroups, the first one composed of current and ex-smokers and the second one composed of nonsmokers. We correlated the expression of TLRs with cigarette smoking. We have not found any significant difference in TLR expression between these two subgroups in our patient groups.

## Discussion

To the best of our knowledge, this is the first attempt to demonstrate that TLRs are implicated in the pathogenesis of fibrotic and granulomatous lung disorders, using BALF, a minimally invasive, useful tool in the everyday diagnostic workup of interstitial lung diseases.

We observed an overexpression of TLR-3 mRNA in patients with fibrotic lung disorders (IPF and CTD-IP) versus patients with nonfibrotic sarcoidosis, suggesting that different activity of this receptor could be one of the mechanisms able to explain the skew toward the Th2 cytokine pattern which characterizes the development of fibrosis. Recently, our study group has proposed distinct angiogenic profiles between IPF and sarcoidosis as another possible mechanism for the development of fibrosis [29,32]. A link between TLR activation and angiogenesis has been observed in patients with Crohn's disease, where activation of TLRs located in gut myofibroblast results in an increased secretion of CXC chemokine ligand 8 (CXCL8), which is a potent angiogenetic factor implicated in the development of fibrosis [33,34].

We also detected an overexpression of TLR-3 mRNA in CTD-IP samples in comparison with IPF. Moreover, knowing that CTD-IPs are a heterogeneous group of diseases, we compared patients with IPF to patients with CTD-IP and radiologic pattern of UIP. We observed upregulation of TLR-3 activity when the UIP pattern is associated with CTDs compared with IPF. It is well known that interstitial pneumonias have a better prognosis when associated with CTDs than in their idiopathic forms, even when they exhibit the same histopathologic pattern [35], reflecting probably distinct pathogenetic pathways. The overexpression of TLR-3 in CTD-IP and CTD-UIP patients could be implicated in this phenomenon. Furthermore, using flow cytometry, TLR-9 was found to be overexpressed in BALF cells of patients with CTD-IP as well as CTD-UIP when compared to IPF. We have recently suggested that an upregulation of angiogenesis in CTD-IPs in comparison with IPF could also be a plausible explanation [36]. Additionally, an overexpression of TLR-7 mRNA in patients with IPF versus healthy subjects was shown, enhancing the possible role of TLRs in the pathogenesis of fibrotic disease.

TLR-3, -7 and -9, all recognizing endosomal ligands, are implicated in the innate immunity, recognizing viral derived RNA and DNA, respectively [7]. Viral infections are thought to represent cofactors in the pathogenesis of autoimmune and idiopathic lung fibrosis. Epstein-Barr virus (EBV) has been isolated from lung tissue of patients with IPF and scleroderma-associated fibrosis [37] and serum complement fixation titers of cytomegalovirus (CMV) were elevated in IPF and CTD-IPs [38], whereas human herpes virus-7 and -8 have also been detected in IPF [39]. Recent data have shown that TLR-9 is upregulated in IPF [12]; thus it is likely that viral infections via activation of TLR-9 could have a key role in the pathogenesis of IPF as well as in the acute exacerbation of the disease observed after an infection. On the other hand, there are no data in the current literature regarding TLR involvement in the pathogenesis of autoimmune lung fibrosis. However, it has been demonstrated that TLRs are involved in the pathogenesis of CTDs via activation through endogenous ligands released during infections [40]. TLR-3 and -9 are expressed in human synovial tissue from RA patients [19-21] and in peripheral blood cells in active systemic lupus erythematosus [18]. Our data demonstrate an increased expression of TLR-3 and -9 in CTD-IPs compared to IPF, suggesting that endosomal TLRs could be a reliable marker in the differentiation of autoimmune from idiopathic fibrosis.

We have also tried to find out if cigarette smoking has some impact on TLR expression. It is of note now that smoking is involved in the pathogenesis of pulmonary fibrosis and influences the outcome [41]. Moreover, smoking and other environmental agents also have a negative effect on innate immunity and various cancers [42]. It has been observed that the expression of TLR-2 and -4 in alveolar macrophage of smokers in response to their ligands is decreased compared to nonsmokers [43]. Additionally, infants of smoking mothers had a significantly attenuated innate TLR-mediated response compared with infants of nonsmokers in monocytes [44]. We have not found any difference in TLR expression when we subdivided the patients of each one of the patient groups into smokers (former and current) and nonsmokers.

However, there are some limitations that need to be addressed. We used BALF to evaluate the activity of TLRs instead of lung tissue. Lung tissue is not warranted for the diagnosis of CTD-IPs, as it does not add information regarding the prognosis of the disease [35] and diagnosis of IPF can be based on imaging findings alone, with the exception of cases exhibiting unusual HRCT findings [24].

## Conclusion

In conclusion, we have demonstrated that dysfunctions of the innate immune system at the TLR level could be implicated in the pathogenesis of fibrotic and granulomatous disorders. TLRs could represent a novel therapeutic target for these disorders and in particular for the most devastating form of fibrosis, IPF. However, further studies are needed to better understand the pathogenetic mechanisms of these complicated diseases.

## Competing interests

The authors declare that they have no competing interests.

## Authors' contributions

GM: participated in the design of the study and wrote the manuscript, KK: carried out the flow cytometry and carried out the real-time-PCR, KDS: helped to draft the manuscript, IL, EV, RL: participated in the sequence alignment and carried out the real-time-PCR, RL: performed the statistical analysis, HK: carried out the flow cytometry, NMS and KMA: participated in conception, coordination and design of the study and helped to draft the manuscript.
